# An Exploratory Research of 18 Years on the Economic Burden of Diabetes for the Romanian National Health Insurance System

**DOI:** 10.3390/ijerph17124456

**Published:** 2020-06-21

**Authors:** Claudiu Morgovan, Smaranda Adina Cosma, Madalina Valeanu, Anca Maria Juncan, Luca Liviu Rus, Felicia Gabriela Gligor, Anca Butuca, Delia Mirela Tit, Simona Bungau, Steliana Ghibu

**Affiliations:** 1 Preclinical Department, Faculty of Medicine, “Lucian Blaga” University of Sibiu, 2A Lucian Blaga St., 550169 Sibiu, Romania; claudiu.morgovan@ulbsibiu.ro (C.M.); ancamaria.juncan@ulbsibiu.ro (A.M.J.); liviu.rus@ulbsibiu.ro (L.L.R.); felicia.gligor@ulbsibiu.ro (F.G.G.); anca.butuca@ulbsibiu.ro (A.B.); 2 Department of Hospitality Services, Faculty of Business, “Babeș-Bolyai” University, 7 Horea St., 400174 Cluj-Napoca, Romania; smaranda.cosma@tbs.ubbcluj.ro; 3 Department of Medical Informatics and Biostatistics, Faculty of Medicine, “Iuliu Haţieganu” University Medicine and Pharmacy, 4 Louis Pasteur St., 400349 Cluj-Napoca, Romania; 4 Department of Pharmacy, University of Oradea, 29 Nicolae Jiga St., 410028 Oradea, Romania; mirela_tit@yahoo.com (D.M.T.); sbungau@uoradea.ro (S.B.); 5 Department of Pharmacology, Physiology and Pathophysiology, Faculty of Pharmacy, “Iuliu Haţieganu” University Medicine and Pharmacy, 4 Louis Pasteur St., 400349 Cluj-Napoca, Romania

**Keywords:** diabetes mellitus cost, physical indicators, efficiency indicators, Romanian National Health Insurance System, National Diabetes Programme, antidiabetic drugs, pharmacoeconomics

## Abstract

The prevalence of diabetes mellitus (DM) rises constantly each year worldwide. Because of that, the funds allocated for the DM treatment have increased over time. Regarding the number of DM cases, Romania is among the top ten countries in Europe. Based on the National Diabetes Programme (NDP), antidiabetic drugs and other expenditures (Self-monitoring blood glucose (SMBG) test, HbA1c, insulin pumps/insulin pumps supplies) are free of charge. This programme has undergone many changes in drugs supply, in the last two decades: re-organizing the NDP, authorization of new molecules with high prices (e.g., SGLT-2 inhibitors, etc.) or new devices (e.g., insulin pumps, etc.) The main purpose of this study is to identify and analyse the impact of the DM costs on the Romanian health budget and to highlight the evolution of these costs. A retrospective longitudinal research on the official data regarding the DM costs from 2000 to 2017 was performed. The DM funds (DMF) were adjusted with the inflation rate. In this period, the average share of DMF in the total funds allocated for health programmes was 21.3 ± 3.4%, and DMF average growth rate was 25.4% (r = 0.488, *p* = 0.047). On the other hand, the DMF increased more than 14 times, in spite of the patients’ number having increased only about 2.5 times. Referring to the structure of DMF, the mean value of the antidiabetic drugs cost was of 96,045 ± 67,889 thousand EUR while for other expenditures it was of 11,530 ± 7922 thousand EUR (r = 0.945, *p* < 0.001). Between 2008 and 2017, the total DMF was 181,252 ± 74,278 thousand EUR/year. Moreover, the average patients’ number was 667,384 ± 94,938 (r = 0.73, *p* = 0.016), and the cost of treatment was 215 ± 36 EUR/patient/year. Even if the cost is rising, the correct and optimal treatment is a main condition for the diabetic patient’s health and for the prevention of its complications, which have multiple socio-economic repercussions.

## 1. Introduction

### 1.1. Background

Worldwide, the prevalence of diabetes mellitus (DM) raised from 135 million people (1995) to 264 million (2006) and 425 million (2017) [1,2]. Furthermore, it is estimated that about 212 million (50%) suffer from DM, without being diagnosed yet. The forecast mentions a figure of about 629 million diabetic patients in 2045. Official data show that the number of patients from low and middle-income countries is very large [1]. 

On the other hand, the number of type 1 diabetic children, diagnosed in 2017, exceeded 1,106,500 [1] (a 42% increase compared to 2011) [2]. The annual incidence increase for this pathology in children reaches 3%, and the number of child patients is today over 490,000. The highest figure of children with type 1 DM can be found in Europe (over 115,000); in Romania, the number of newly diagnosed children ranges between 5 and 8.5 per 100,000 children [2]. 

The DM leads to a diminution of life quality due to both acute and chronic consequences, reduces life expectancy and leads to higher mortality [3,4,5,6]. DM caused 4.0 million deaths in 2017. It is among the five most common causes of death in most high-income countries [1,2]. In 1989, the St. Vincent Declaration (Italy), a joint act of International Diabetes Federation (IDF) together with many EU countries and the National Associations of Diabetic Patients, was produced. According to this act, the implications of DM for people’s health are very important. All the authorities have to collaborate to find solutions to prevent, treat, control and monitor this pathology [7,8]. DM caused at least USD 727 billion dollars in healthcare expenditures in 2017, representing 12% of total spending on adults [1]. Developed countries allocate 5–10% of the health budget for diabetic care. For example, in 1997, in Japan, the cost of DM was 8 billion USD (4% from Health budget) [9].

In the USA, between 1998 and 2017, the total expenses with DM had a fourfold increase up to 327 billion USD [10,11,12,13]. In Canada, the DM expenses increased with 0.5 billion USD, from 1993 to 1998. [14]. Another study shows that in 1978 Sweden allotted for DM treatment only 291 million USD (1.5% from Health funds), while in 1994, 744 million USD (2.1% from Health funds) [11]. Nowadays, some EU states like Germany, France, Italy, the United Kingdom, Spain and Denmark spend annually over 1 billion EUR for DM treatment [15]. In some studies (e.g., Canada, Romania, Latin America and the Caribbean, etc.) it is noticed that the complications or hospitalization can increase the cost of therapy by 200–400% [14,16,17]. Barcelo et al. [17] estimated that the cost of DM in Latin America and the Caribbean was of 65.216 billion USD. They considered that direct costs were about 16%, and the early retirement and disabilities had a higher economic impact. Other studies show that complications increase the costs between 10% and 31.4%, in France [18,19,20,21], or even up to 60%, in Japan [9]. The “Cost of Diabetes in Europe-Type II” study showed that the cost of DM treatment is about 3000 EUR/patient/year (21.7% drugs, 59.8% hospitalization, 18.5% outpatient care) [22]. 

**Figure 1:**
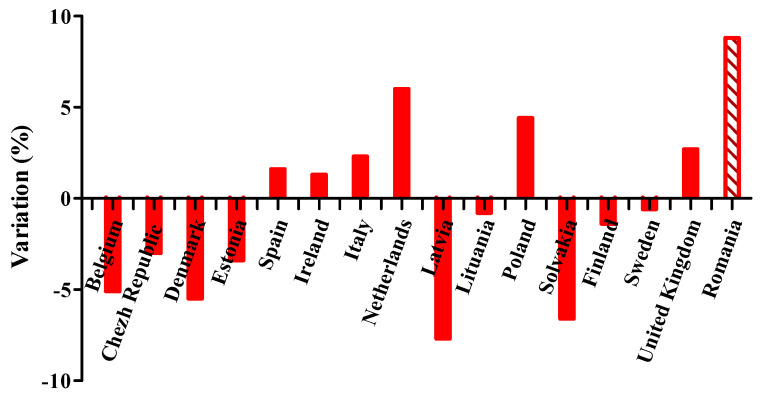
Variation of the percentage of diabetes funds in the total health expenditure in 16 EU countries (2011 vs. 2007).

From the data presented by Directorate c–Public Health in the European Commission–Health and consumers directorate-general [15,23], it can be observed that the percentage of diabetes funds in the total health expenditure differs greatly from country to country. Figure 1 shows the variation of this share in 2011 compared to 2007, in 16 EU countries. From all these countries, the best evolution was for Romania, from 2.0% to 10.8% (+8.8%), where the share of DM in the health budget increased more than 4 times.

In Romania, in the fourth trimester of 2007, only some months after running the national programme for the evaluation of the population state of health (June 2007), out of 2,752,953 people evaluated, 825,540 (30%) presented risks of DM [24,25]. The statistical data regarding the prevalence of DM among adult population in 2012 ranked Romania (9.3%) above the global average (8.3%) [2]. In 2018, the official data included more than 860,000 registered patients with DM [26] and according to IDF, Romania was among the first top ten countries with DM cases in Europe (prevalence is 12.4%) [1]. At the moment, in Romania, the number of patients (diagnosed and undiagnosed) is estimated over 1.5 million [1]. 

The Romanian diabetic patients are recorded by the diabetologists in National Diabetes Programme (NDP). The diagnostic codes eligible for this health programme are classified according to the International Statistical Classification of Diseases and Related Health Problems 10th Revision (ICD10): Insulin-dependent DM (code 241), Non-insulin-dependent DM (code 242), Malnutrition-related DM (code 243), Other specified DM (code 244) and Unspecified DM (code 245). According to Romanian rules, the direct cost of DM is free of charge for patients and includes as follows: (a) antidiabetic drugs (oral antidiabetic drugs and insulins), (b) other expenditures (Self-monitoring blood glucose (SMBG) test, glycated haemoglobin assessment-HbA1c, insulin pumps or insulin pumps supplies). The second category is free of charge only for patients treated with insulin [26,27,28]. 

In Romania, ethical drugs (Rx), including antidiabetic drugs, can be purchased by patients on the base of the doctor’s prescription, at a maximum price approved by the Ministry of Health. Between 2000 and 2017, in Romania, the patients could purchase the antidiabetic drugs, free of charge, but the NDP has performed differently [8,29,30,31,32]:

- Until 2003, the drugs could be purchased from some pharmacies (hospital or community), and the Romanian National Health Insurance House (RNHIH) discharged their costs without any compulsory reduction from the maximum price approved by the Ministry of Health.
- Between 2003 and 2007, the antidiabetic drugs were discharged by RNHIH, at a price established on base of the national bidding. In this period, only a few hospital pharmacies were included in NDP for supplying related drugs.
- During 2007-2011, the antidiabetic drugs could be acquired from community pharmacies. The cost discharged by the RNHIH must be reduced according to the negotiations organized by RNHIH.
- After 2012, antidiabetics can be purchased from community pharmacy, and RNHIH reimburses the total cost of these drugs, which is the maximum price of generics approved by the Ministry of Health.

After the decentralization of the NDP to community pharmacies (2007), RNHIH could not reimburse all costs for the medication in the same year. Therefore, a part of these amounts was paid in the following year. In 2012 and 2013, RNHIH reimbursed to pharmacies a part of the older debts.

### 1.2. Objectives

In Romania, the data regarding the evolution of the number of diabetics or DM cost is not centralized. Moreover, there are no public studies which assess the economic impact of DM treatment on the heath budget. Filling this gap, the present paper offers an overview and an analysis of the evolutionary situation from Romania, in terms of DM, considering the pharmacoeconomic aspects. This research is focused on identifying the DM pressure on the Romanian health budget and the evolution of these costs over almost two decades, taking into account that DM is among the first fifth pathologies as cost of chronic diseases and one of the most cost-generating diseases included in the Romanian National Health Programmes. 

For the purpose of this study, the following research objectives were formulated:

1. Studying the diabetes mellitus funds (DMF) evolution as part of the total cost of National Health Programmes (NUFSHI HP);
2. Analysing the structure of DMF (medication and other expenses);
3. Identifying the physical and efficiency indicators and their evolution over time;
4. Identifying the possible correlations between these variables.

## 2. Materials and Methods 

### 2.1. Study Design

In this paper an exploratory research was performed using the secondary data analysis as research method. The Romanian regulations concerning DM in the period 2000–2017 and the official data regarding the DM costs were analysed. Logical analysis and comparison were used as work techniques [33]. We realized a longitudinal retrospective study. To carry out this study, data were collected from RNHIH annual reports. The chronic diseases with major socio-economic impact are included in the National Health Programmes (DM included), and the funds are allotted by RNHIH. The total funds for these programmes are centralized as the National Unique Fund of Social Health Insurance for Health Programmes (NUFSHI HP) [24,26]. 

### 2.2. Setting

For each year, we extracted data from the RNHIH reports where the structure of NUFSHI HP is detailed, considering for analysis physical indicators (number of diabetic patients, number of patients who benefited from HbA1c assessment, SMBG tests, insulin pumps or supplies for insulin pumps) and efficiency indicators (NUFSHI HP, funds allotted annually for diabetic patients, cost of DM treatment/patient/year, cost of HbA1c assessment, cost of SMBG test, cost of insulin pump, cost of supplies for insulin pumps). All indicators analysed in the present study are established by the Romanian legislation [24,26,27,32,34]. First, the evolution of NUFSHI HP, during 2000–2017 was analysed. For a higher accuracy, these values have been adjusted with the inflation rate each year, and the adjusted growth for NUFSHI HP was properly identified. The annual inflation rates calculated by the Romanian National Statistics Institute using the *Consumer Price Index* [35] *were used*. Moreover, the evolution of the funds allocated for the diabetes mellitus programme (DMF) was analysed for the interval 2000–2017; these values have been updated with the inflation rate to obtain the adjusted growth of DMF. Later, both series have also been compared. Data regarding all indicators are available only for the last ten years of the study. For this reason, the physical (number of patients) and efficiency indicators (average cost/patient/year, cost of HbA1c/assessment, cost of SMBG test, cost of insulin pump and cost of supplies for insulin pumps) have been analysed only for 2008–2017. 

All costs were calculated in euros, taking into account the average exchange rate of the Romanian National Bank, for each year of the period 2000–2017 [36]. 

### 2.3. Statistical Methods

All data entry and management activities were undertaken on a GraphPad spread sheet. The SPSS program for Windows version was used to analyse the data. Data are expressed as mean ± standard deviation, and the Pearson or Spearman correlation coefficient was calculated (according with data type). The acceptable error threshold was *p* < 0.05. Colton rules and significance test were used to interpret the correlations [37,38,39].

## 3. Results

### 3.1. Outcome Data-Evolution of DMF in Romania during 2000–2017

**Figure 2:**
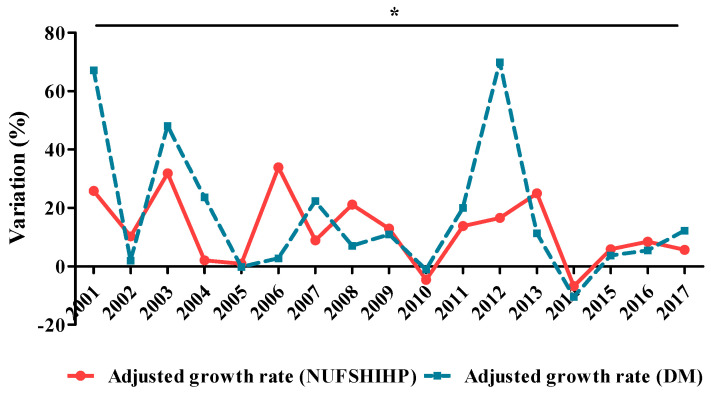
Adjusted growth rate of diabetes mellitus (DM) funds (DMF) compared to National Unique Fund of Social Health Insurance for Health Programmes (NUFSHI HP) (2000–2017). (r = 0.488, * *p* = 0.047).

Figure 2 presents the evolution of the adjusted growth rate of NUFSHI HP and DM.

It can be observed that, NUFSHI HP and DMF had a constant increase during the analysed period. The average share of NDP from total NUFSHI HP in 2000–2017 was 21.3 ± 3.4% (maximum in 2012—28.3% and minimum in 2000—15.3%). The average growth rate of DMF was 25.4%, and the most important increases were 101.7% (2001), 73.2% (2012) and 63.3% (2003). It has to be mentioned that in 2014, DMF had a negative rate (−9.4%). The adjusted growth rate of NFUSHI HP had mean values of 18.1 ± 26.7% and the adjusted growth rate of DMF averaged 17.3 ± 23.3%. Both data series have a similar growth rate over the period analysed, with a good, direct proportional dependence, with statistical significance (Spearman correlation coefficient r = 0.488, * *p* = 0.047). 

**Figure 3:**
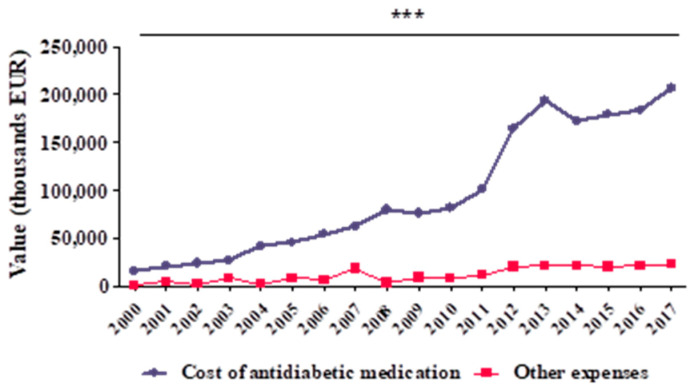
Structure of diabetes DMF during 2000–2017 (thousand EUR): cost of antidiabetic medication (OADs and insulins) and other expenses (HbA1c assessment, Self-monitoring blood glucose (SMBG) test, insulin pumps and supplies for insulin pumps) (r = 0.945, *** *p* < 0.001).

Figure 3 presents the structure of the DM cost (thousand EUR) between 2000 and 2017. This indicator includes two cost categories: cost of antidiabetic medication (oral agents - OADs and insulins) and cost of other expenses (HbA1c assessments, SMBG, insulin pumps and supplies for insulin pumps).

The total cost of antidiabetic drugs had mean values of 96,045 ± 67,889 thousand EUR and other expenditure had mean values of 11,530 ± 7,922 thousand EUR. In 2000–2017, the average share of other cost expenditures in DMF was 13.0% (minimum: 4.6% in 2008; maximum: 30.6% in 2003). There is a very good, directly proportional correlation, with statistical significance (r = 0.945, *** *p* < 0.001) between the total cost of antidiabetic drugs and other expenditures (Figure 3).

### 3.2. Main Results—Physical Indicators

#### 3.2.1. Number of Diabetic Patients

**Figure 4:**
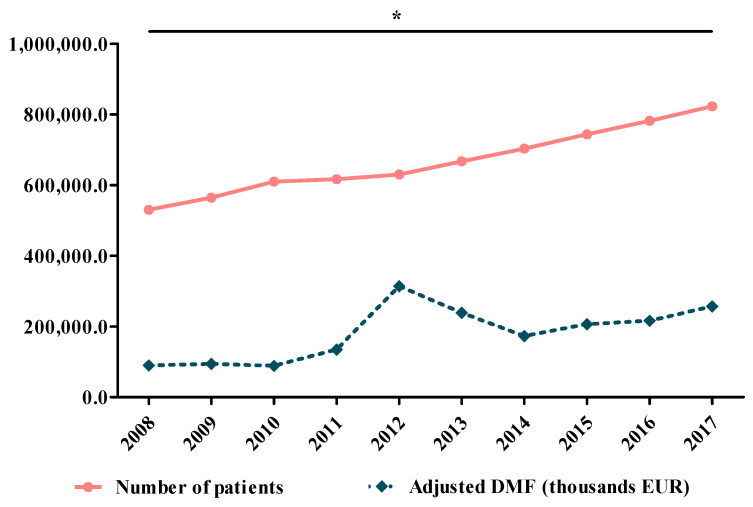
Evolution of the number of patients compared to the adjusted value of DMF (thousand EUR) (r = 0.73, * *p* = 0.016).

Figure 4 shows the evolution of the patients’ number compared to the adjusted value of DMF (thousand EUR).

In the investigation period, in Romania, the number of diabetic patients increased with 55.2% from 530,482 (2008) to 823,271 (2017). The average annual growth rate of the number of patients (2008–2017) was 6.1% (minimum: 1.1% in 2011; maximum 15.9% in 2008). Total diabetic patients averaged 667,384 ± 94,938 and total DM cost (adjusted with inflation) had mean values of 181,252 ± 74,278 thousand EUR. There is a good correlation, directly proportional between patients’ number and DMF, with statistical significance (r = 0.73, * *p* = 0.016).

#### 3.2.2. Number of Patients Who Benefited from HbA1c Assessments, SMBG Tests, Insulin Pumps or Supplies for Insulin Pumps

**Figure 5:**
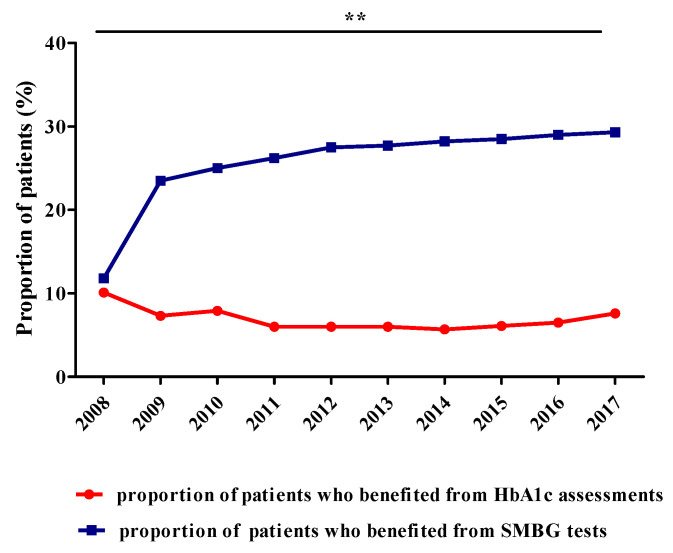
Evolution of the number of patients who benefited from HbA1c assessments or SMBG tests (adults and children) (r = −0.781, ** *p* = 0.008).

Figure 5 presents the evolution of the patients’ number who benefited from HbA1c assessments or SMBG test (adults and children).

Annually, the HbA1c assessments were provided free of charge for about 49,585 ± 15,210 patients treated with insulin. The percentage of patients that had HbA1c free of charge had mean values of 6.9 ± 1.4%. On the other hand, the number of patients (adults and children) who benefited free of charge from SMBG test increased about 4 times, from 62,604 in 2008 to 241,591 in 2017. The proportion of SMBG patients (children + adults) had mean values of 25.7 ± 5.2% (Figure 5). Therefore, it is observed that there is a very good, inversely proportional dependence between the proportion of patients who benefited from HbA1c and those who benefited from SMBG tests, with statistical significance (r = −0.781, ** *p* = 0.008). 

The number of patients with insulin pumps increased from 14 (2013) to 279 (2017), and the number of patients who received supplies for insulin pumps increased from 168 (2013) to 506 2017).

### 3.3. Main Results—Efficiency Indicators

**Figure 6:**
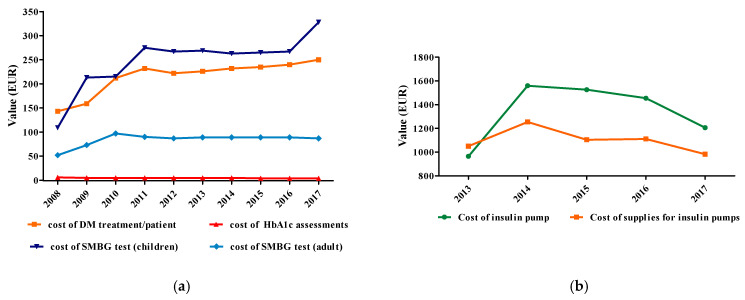
Efficiency indicators (EUR): (**a**) cost of DM treatment/patient/year (EUR), cost of HbA1c assessments (EUR), cost of SMBG test for children (EUR), cost of SMBG test for adult (EUR), (**b**) cost of insulin pump (EUR), cost of supplies for insulin pumps (EUR).

NDP included the following efficiency indicators: (1) cost of DM treatment/patient/year, (2) cost of HbA1c assessment, (3) cost of SMBG test, (4) cost of insulin pump, (5) cost of supplies for insulin pumps/year (Figure 6).

#### 3.3.1. Cost of DM Treatment/Patient/Year

During 2008–2017, in Romania, the cost of treatment/patient/year had an increasing trend, from 143 to 250 EUR (215 ± 36 EUR/patient/year) (Figure 6a).

#### 3.3.2. Cost of HbA1c Assessment 

The number of patients that benefit from medication has increased a lot, compared to patients who received self-monitoring tests or HbA1c testing. During 2000–2008, the cost of HbA1c assessment (Figure 6a) had a constant evolution of 4.9 ± 0.5 EUR.

#### 3.3.3. Cost of SMBG Test

The cost of SMBG test for adults had a constant evolution of 90 ± 3 EUR. For children, in 2008, a low cost of SMBG tests of 109 EUR was noticed. The cost of child tests had mean values of 247 ± 58 EUR, while adult test costs averaged 84 ± 13 EUR. There is a very good, directly proportional correlation dependence between the two costs (Figure 6a), with statistical significance (r = 0.854, ** *p* = 0.002). For 2009–2010 this cost has doubled and in 2011–2017 has been triple or more up to 328 EUR.

#### 3.3.4. Cost of Insulin Pumps/Patient and Cost of Supplies for Insulin Pumps/Patient/Year

The annual cost of insulin pumps per patient included in the Romanian official data varied between 964 EUR in 2013 and 1559 EUR in 2014. Moreover, the supplies for insulin pumps had an increased cost, which fluctuated between 983 EUR in 2017 and 1254 EUR in 2014 (Figure 6b). The correlation between the cost of insulin pumps and insulin pump supplies is good, directly proportional, but not statistically significant (*p* > 0.05). 

### 3.4. Other Analyses-Association between DMF (EUR) and Other Indicators

**Figure 7:**
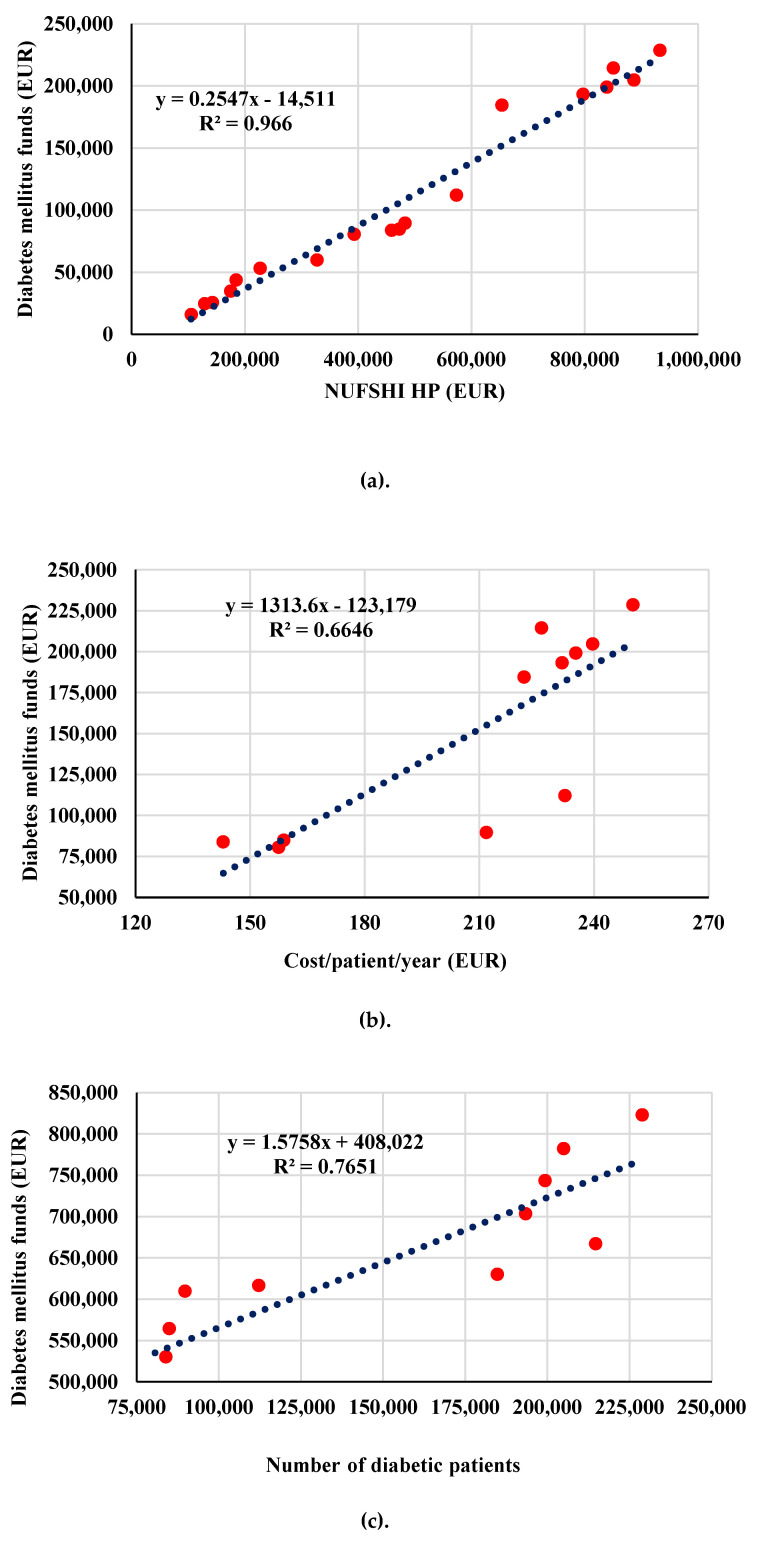
Association between DMF and NUFSHI HP (**a**), Cost/patient/year (**b**) and Number of patients (**c**).

DMF indicator (EUR) was associated with total funds allotted for Romanian National Health Programs (Figure 7a). There is a very good direct association between both indicators (r = 0.98, *** *p* < 0.001).

An association (r = 0.815, *** *p* < 0.001) between diabetes mellitus funds (DMF) and cost/patient/year (Figure 7b) and a better association (r = 0.87, *** *p* < 0.001) between DMF and number of diabetic patients (Figure 7c) can be observed.

## 4. Discussion

This study analyses the information regarding the public funds allocated for DM treatment in Romania, between 2000 and 2017. The DM treatment can be prescribed by diabetologists, general practitioners or internal medicine physicians. According to the Order of Health Ministry no. 1061/425/2006, the drugs can be purchased from public pharmacies and are free of charge for all patients [32]. The DM Romanian national programme aimed at improving the health of the diabetic people, by providing them with access to care, laboratory tests and self-monitoring, to increase their life expectancy. The main activities financed from the allocated budget are [24,32,40]: information, education, communication and continuous staff training; SMBG of insulin-treated patients; access to insulin pumps and anti-diabetic care (oral antidiabetics and insulins); control of DM patients by periodic check-ups of HbA1c. 

As the number of newly diagnosed patients increases annually and medication from new structural classes and chemical molecules appear [18], but at a higher cost, the funds required also increase [31,41,42]. Between 1 July 2007 and 31 December 2008, a national programme assessing people’s health status was implemented. Thus, the DM patients’ number increased significantly in 2008 (more than 15%) compared to 2007 [8,32]. Since 2009 when this programme finished, the number of patients has increased to 823,271 (2017), by only 5.0 ± 2.1%/year, which demonstrates that an important number of undiagnosed people can still be found. On the other hand, we can take into account that in order to increase DM control, some of the patients need a constant intake of insulin during the entire day. This control can be obtained by self-monitoring, periodic evaluation of HbA1c or using insulin pumps which deliver insulin subcutaneously, throughout the day [43]. The present study also shows that between the evolution of SMBG tests and HbA1c assessments there is a very good, inversely proportional correlation (r = 0.781, *p* = 0.008). Although all patients treated with insulin have to benefit from SMBG and HbA1c, only a small part of them benefited from HbA1c assessments free of charge. Therefore, according to Figure 5, maximum 10.1% from total patients had benefited from HbA1c assessment and maximum 29.3% from SMBG tests. Moreover, it must be emphasized that the cost of HbA1c assessments is small (4.9 ± 0.5 EUR). Referring to the insulin pumps, their costs are free of charge, and the first official data regarding this device are available starting with 2013. During the period 2013–2017, the number of patients with insulin pumps increased from 14 to 279 [26].

In Romania, DMF represent more than a fifth (21.25 ± 3.4%) from total funds intended for health programmes. The reimbursement of the DM cost at maximum price approved by the Ministry of Health and the payment of older debts, generated in 2012 and 2013 a deviation of DMF from its trend. Overall, the DMF (EUR) increased more than 14 times, although the number of patients increased about only 2.5 times. This gap shows that the services supplied improved qualitatively due to the following issues: higher numbers of self-monitored patients; larger number of specialists monitoring the evolution of the diabetic patients (diabetologists and general practitioners); the access of more patients to HbA1c tests and SMBG tests [26,27,28,30]; the access of the patients to the most recent medication, with increased price and quality (e.g., insulin analogues and combinations, thiazolidinediones, GLP-1 analogues, DPP-4 inhibitors or SGLT-2 inhibitors and fixed combinations, etc.), as well as to modern pharmaceutical forms or devices (prefilled insulin pens, modified or prolonged release, gastro-intestinal therapy, insulin pumps) with costs higher than for conventional treatment etc. [8,31,42]. 

As presented above, DMF are allocated only for a few types of expenses, not for complications or hospitalization, which can generate high costs. On the other hand, the productivity loss can also represent important shares of total DM costs (41%), compared to other drugs (4%) [44,45]. In this study, the share of antidiabetic drug cost (96,045 ± 67,889 thousand EUR) represented about 88.5% from total funds allotted for diabetes. In 1998, CODE-2 research (1988–1998) was performed in eight EU countries, emphasizing that the cost of antidiabetic drugs represented only 7% from total DM cost [46]. Recently, in a research made in 2012, in France, the cost of antidiabetic drugs represented 49% for all diabetes-specific expenditure (2.3 billion EUR) [47].

Regarding the efficiency indicators analysed in our study, it can be noticed that the cost of DM/patient/year increased from 143 EUR to 250 EUR (Figure 6). This value includes only the cost of antidiabetic drugs (OADs and insulins). Compared to Romania, in 2002, in France, the DM cost was estimated to be 3,914 EUR/patient/year [19,20,21]. A study made in Japan, in 1997, showed that the direct cost was 21,121 USD/patient/year [9]. In Germany, in 2004, the cost of antidiabetic drugs/patient/year (559 EUR) grew up to 60% compared to 1994 [48]. Seuring et al. [49] identified that direct costs/patient/year as ranging from 242 USD (in Mexico) to 11,917 USD (in USA). Other studies conducted in lower-middle income countries showed an important variation of the annual average cost/patient (29.91–237.38 USD) [50]. All these data highlight the need to properly monitor the disease and emphasize the importance paid to this pathology by the governments of various states by allotting huge funds to prevent, treat and monitor the disease and to diminish additional costs.

Improving the provided services quality of the healthcare system in Romania (including increased system financing) will generate higher patients’ satisfaction and implicitly their improving treatment adherence [51].

Our study shows that the total funds allotted for DM treatment were direct proportionally with total funds allotted by the Romanian authorities for National Health Programmes during the analysed period. Moreover, a direct association can be noticed between the DM funds allotted and cost for every patient by year and also between DM funds and the number of diabetic patients. 

Based on the official data from the years 2000–2017, we estimated the DM financing for the next years in Romania using the trend and forecast functions from Microsoft Excel. Thus, excluding other determinants, in 2025, the total funds allocated by the Romanian authorities could increase by 30% to 336,000 thousand EUR. Nevertheless, this estimated value may not be confirmed, taking into account various corrections that could not be anticipated at this time.

These adjustments could be generated by different needs and not by the real costs. For example, a similar situation was presented above for 2012 and 2013. Additionally, during the analysed period, a lot of new molecules or medical devices (e.g., SGLT-2 inhibitors, insulin pumps, etc.) were introduced in diabetes treatment. The new therapies have higher costs and their reimbursement is randomly established by authorities. This kind of aspects could cause deviations from predictions made.

The data above show that during the last ten years of the study, the amounts spent with DM increased together with the number of patients benefiting from health care; the quality of services furnished increased as more doctors and better trained personnel joined this effort. Following free of charge self-monitoring, HbA1c assessments or insulin pumps, new suppliers with higher quality products entered the market. All these prove that the funds allocated to DM were used both to supply medication and materials for the treatment and monitoring of the disease for a larger number of patients, as well as the fact that the services provided had a higher cost and also a better quality. Nowadays, the economic evaluation is more and more common for various treatments [52]. The correct treatment administered in time is the main condition for the diabetic patient’s health, being essential for the prevention of the diabetes mellitus complications. A bad patient condition may require extra care or hospitalization [53,54,55], and also, it can also lead to early retirement or even early mortality. All these represent additional expenditures from the budget of social insurance. 

Different studies are presented the burden of diabetes complications on the healthcare budget [53,54,55]. Di Giovanni et al. highlighted that diabetic foot disease has higher costs and leads to the worst consequences, as well as lower extremity amputation (LEA) [53]. These worsen the quality of life and increase the cost [54,55]. Taking into account that patients with major amputations have more than 3 comorbidities (adjOR 2.51, 95%CI: 1.75–3.60) and those with minor amputation have 2 comorbidities (adjOR 0.51, 95%CI: 0.42–0.64) [53], the cost of diabetes can be much higher. In a study published in 2016, Bondor et al. showed that in Romania, 3.5% of diabetic patients suffered a LEA [55]. In another study, conducted between 2006 and 2010, in all hospitals from Romania, the annual number of amputations was 4,584.4 ± 612.42 [56]. Because of the higher frequency of LEA among the young people, DM is a major healthcare problem. Thus, the authors emphasized the need to implement different program for screening and patients’ education in diabetes foot care [55] and to elaborate national guidelines [54,55].

In addition to a higher number of patients treated, the implementing of the NDP has led to a better life quality and to a higher degree of patients’ satisfaction [57]. 

Our study could support authorities to identify some shortcomings of funding the National Diabetes Programme. A deeper analysis could be made if the funds would mainly include other costs (e.g., hospitalization) or if they would be structured according to the DM type. All these improvements should lead to a better management and control of the DM funds. 

Regarding the DM prevention, Romanian National Public Health Institute together with professional associations elaborated educational materials and financed various campaigns or studies (e.g., PREDATORR) in order to analyse the behavioural determinants and their impact for diabetes mellitus [58,59,60]. Although the authorities allocate different funds for promoting the healthy lifestyle or for sustaining the screening tests, for more efficiency, these measures should be included in the National Diabetes Programme.

Although our study provides important information about the evolution of DMF during a significant period, a limitation of the study refers first to the impossibility of analysing information structured on both types of DM, because the official documents published by the Romanian authorities do not consider this classification of DM. In addition, our study cannot consider the cost of DM complication or hospitalization in Romania, because this data is not available either. 

## 5. Conclusions

To our knowledge, this research presents for the first time a long-term analysis of the DM pressure on the Romanian health budget and the evolution of these costs over almost two decades. The study evaluates physical and efficiency indicators established by the National Diabetes Programme. The obtained results of our research could be an important support for the institutions involved, to generate policies or strategies for the health system or to help use public funds more efficiently. In Romania, as in other countries, the disease incidence is worrying because the number of patients doubled in the investigated interval. Over time, the National Diabetes Programme has had many changes, which were reflected on the increasing evolution of funds allocated to diabetes, as well as on efficiency indicators analysed. The long-term control of the diabetic disease has as result the reduction of the risks of complications and, implicitly, of the costs. An important objective for the Romanian health authorities should be the reinsertion of the National programme for the evaluation of population’s health (stopped in 2009), through which the state of health could be monitored, including the risks of diabetes mellitus. The present study could be a starting point for another analysis in the field of healthcare.

## Abbreviations

DM	Diabetes mellitus
DMF	Diabetes mellitus fund
DPP-4	Dipeptidyl peptidase-4
GLP-1	Glucagon-like peptide-1
HbA1c	Glycated haemoglobin
IDF	International Diabetes Federation
NDP	National Diabetes Program
NUFSHI HP	National Unique Fund of Social Health Insurance for Health Programmes
OADs	Oral antidiabetic drugs (including GLP-1 analogues)
RNHIH	Romanian National Health Insurance House
SGLT-2	Sodium-glucose co-transporter-2
SMBG	Self-monitoring blood glucose
